# Antimicrobial Activity of Auranofin, Cannabidivarin, and Tolfenamic Acid against Multidrug-Resistant Neisseria gonorrhoeae

**DOI:** 10.1128/spectrum.03952-22

**Published:** 2022-11-09

**Authors:** Fan Yang, Jin Liu, Yuhua Gu, Ruilin Jiao, Jing Yan, Shuai Gao, Xu’ai Lin, Stijn van der Veen

**Affiliations:** a Department of Microbiology, and Department of Dermatology of Sir Run Run Shaw Hospital, School of Medicine, Zhejiang University, Hangzhou, People’s Republic of China; b State Key Laboratory for Diagnosis and Treatment of Infectious Diseases, Collaborative Innovation Center for Diagnosis and Treatment of Infectious Diseases, The First Affiliated Hospital, School of Medicine, Zhejiang University, Hangzhou, People’s Republic of China; c Zhejiang Provincial Key Laboratory for Microbial Biochemistry and Metabolic Engineering, Hangzhou, People’s Republic of China; University of Guelph

**Keywords:** *Neisseria gonorrhoeae*, auranofin, cannabidivarin, tolfenamic acid, FC428

## Abstract

Alternative antimicrobial therapies are urgently required for the multidrug-resistant bacterial pathogen Neisseria gonorrhoeae, for which currently ceftriaxone is the only remaining recommended first-line therapy. Repurposing of drugs that are approved for other clinical applications offers an efficient approach for development of alternative antimicrobial therapies. Auranofin, cannabidivarin, and tolfenamic acid were recently identified to display antimicrobial activity against N. gonorrhoeae. Here, we investigated their activity against a collection of 575 multidrug-resistant clinical isolates. All three compounds displayed consistent antimicrobial activity against all isolates, including against strains associated with the high-level ceftriaxone-resistant FC428 clone, with both the mode and MIC_90_ for auranofin of 0.5 mg/L, while both the mode and MIC_90_ for cannabidivarin and tolfenamic acid were 8 mg/L. Correlations between MICs of ceftriaxone and auranofin, cannabidivarin or tolfenamic acid were low, indicating that development of cross-resistance is unlikely. Furthermore, antimicrobial synergy analysis between ceftriaxone and auranofin, cannabidivarin, or tolfenamic acid by determination of the fractional inhibitory concentration index (FICI) resulted in an interpretation of indifference. Finally, time-kill analyses showed that all three compounds are bactericidal against both the N. gonorrhoeae ATCC 49226 reference strain and an FC428-associated clinical isolate, with particularly cannabidivarin displaying rapid bactericidal activity. Overall, auranofin, cannabidivarin, and tolfenamic acid displayed consistent antimicrobial activity against multidrug-resistant N. gonorrhoeae, warranting further exploration of their suitability as alternative antimicrobials for treatment of gonococcal infections.

**IMPORTANCE** Neisseria gonorrhoeae is a major public health concern because of the high incidence of gonorrhea and the increasingly limited options for antimicrobial therapy. Strains associated with the FC428 clone are a particular concern because they have shown global dissemination and they display high-level resistance against the currently recommended ceftriaxone therapy. Therefore, development of alternative antimicrobial therapies is urgently required to ensure treatment of gonorrhea remains available in the future. Repurposing of clinically approved drugs could be a rapid approach for the development of such alternative antimicrobials. In this study, we showed that repurposing of auranofin, cannabidivarin, and tolfenamic acid for antimicrobial therapy of gonorrhea deserves further clinical explorations because these compounds displayed consistent antimicrobial activity against a large collection of contemporary multidrug-resistant gonococcal isolates that included strains associated with the FC428 clone.

## INTRODUCTION

Neisseria gonorrhoeae is a multidrug-resistant human-specific bacterial pathogen (1), which requires urgent development of novel or alternative antimicrobial agents due to the rise in resistance against ceftriaxone, the currently last remaining recommended first-line antimicrobial therapy (1). Recent years have demonstrated increasing incidences of ceftriaxone-treatment failure (2–4), with a particular threat posed by strains associated with the high-level ceftriaxone-resistant FC428 clone or containing its mosaic *penA* allele 60.001, which has shown global dissemination (5–8) and have become widespread in China (9–11). Recent clinical trials for alternative clinically approved antimicrobials such as gentamicin and fosfomycin have largely been unsuccessful (12, 13), with the possible exception of ertapenem (12). Importantly, strains associated with the FC428 clone appeared to be susceptible to ertapenem (11, 14, 15).

To broaden the search for effective antigonococcal compounds, repurposing and screening of drugs approved for other clinical conditions is an effective approach and has resulted in the identification of antigonococcal activity for auranofin (16), cannabidivarin (17), and tolfenamic acid (18). Auranofin is an antirheumatic drug that has displayed good antimicrobial activity against multidrug-resistant Gram-positive bacteria such as Staphylococcus aureus, Streptococcus pneumoniae, and *Entercoccus faecalis* (19–21) and against Mycobacterium tuberculosis (20), yet, except for N. gonorrhoeae, not against Gram-negative bacteria due to limited penetration of the outer membrane (16, 20, 21). Clinical studies on cannabinoids have thus far attributed a variety of properties, including anti-inflammatory and neuroprotective activities (22, 23), but more importantly, antimicrobial properties against particularly Gram-positive bacteria have also been described (24, 25). However, a recent screen of antimicrobial activity by cannabidiol-derivatives identified activity against N. gonorrhoeae, with the most potent activity displayed by cannabidivarin (17). Tolfenamic acid is a nonsteroidal anti-inflammatory drug that inhibits the production of prostaglandins (26). Recently it was shown to display consistent activity against a small collection of N. gonorrhoeae clinical isolates (18). Here, we investigated the activity of auranofin, cannabidivarin, and tolfenamic acid against a collection of recent multidrug-resistant clinical isolates that include strains associated with the FC428 clone.

## RESULTS

### Gonococcal susceptibility to auranofin, cannabidivarin, and tolfenamic acid.

**FIG 1:**
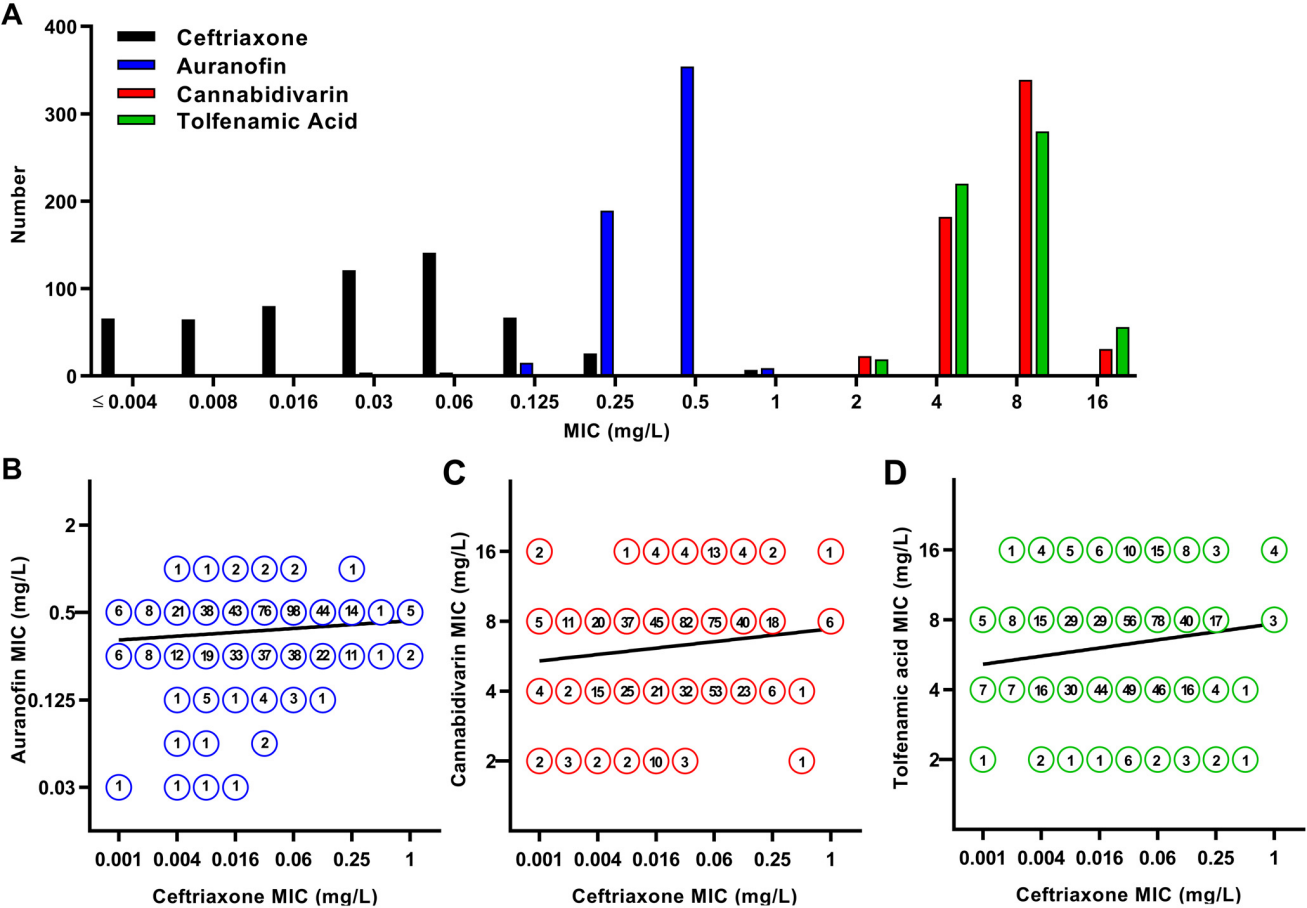
Gonococcal susceptibility to auranofin, cannabidivarin, and tolfenamic acid and their susceptibility correlation analysis with ceftriaxone. (A) Distribution of MICs for 575 clinical Neisseria gonorrhoeae isolates. (B) Correlation between MICs of auranofin and ceftriaxone. (C) Correlation between MICs of cannabidivarin and ceftriaxone. (D) Correlation between MICs of tolfenamic acid and ceftriaxone. Each symbol in panels b to d represents one or more isolates, indicated by a specific number within the symbol. The regression line is shown.

The gonococcal susceptibility to auranofin, cannabidivarin, and tolfenamic acid and reference antimicrobials was investigated for 575 clinical isolates (Table 1). These clinical isolates show high incidences of resistance against the reference antimicrobials, including 6% resistance for ceftriaxone (MIC > 0.125 mg/L) and nine isolates associated with the FC428 clone, and 20% resistance to azithromycin (MIC > 0.5 mg/L). Based on absolute MIC levels for the three tested alternative compounds, auranofin showed the most potent antimicrobial activity, with both a mode and MIC_90_ of 0.5 mg/L, while both the mode and MIC_90_ for cannabidivarin and tolfenamic acid were 8 mg/L. Histograms of MIC levels were relatively narrow for all three compounds (Fig. 1A), with 94% of the isolates displaying an auranofin MIC of 0.25 to 0.5 mg/L. Similarly, 91% of the isolates showed a cannabidivarin MIC of 4 to 8 mg/L and for tolfenamic acid 87% of the isolates showed an MIC of 4 to 8 mg/L. For comparison, the mode and MIC_90_ for ceftriaxone were 0.03 mg/L and 0.125 mg/L, respectively. Fortunately, the likelihood for cross-resistance development between ceftriaxone and auranofin, cannabidivarin, or tolfenamic acid is low, given that the ceftriaxone MICs were poorly correlated with the MICs of auranofin (Fig. 1B; *R* = 0.13), cannabidivarin (Fig. 1C; *R* = 0.14), and tolfenamic acid (Fig. 1D; *R* = 0.16).

**TABLE 1:** Antimicrobial susceptibility of 575 Neisseria gonorrhoeae clinical isolates

Antimicrobial	MIC range (mg/L)	Mode (mg/L)	MIC_90_^*a*^ (mg/L)
Auranofin	0.03 to 1	0.5	0.5
Cannabidivarin	2 to 16	8	8
Tolfenamic acid	2 to 16	8	8
Ceftriaxone	0.001 to 1	0.03	0.125
Cefixime	0.001 to 2	0.06	0.25
Penicillin	0.03 to 2048	4	64
Tetracycline	0.008 to 128	2	64
Ciprofloxacin	0.25 to 128	16	32
Azithromycin	0.001 to 2048	0.125	256
Spectinomycin	1 to 64	32	32

a MIC_90_, MIC that inhibits 90% of the isolates.

### Auranofin, cannabidivarin, and tolfenamic acid antimicrobial synergy testing with ceftriaxone.

Besides single antimicrobial therapy, antimicrobials can also be used in combination as a dual antimicrobial therapy when treatment security of single antimicrobials is suboptimal or to prevent development of resistance against single antimicrobials. Given that ceftriaxone is currently the recommended treatment for gonorrhea, antimicrobial synergy was tested for auranofin, cannabidivarin, and tolfenamic acid combined with ceftriaxone (Table 2). The FICI for all three compounds with ceftriaxone was 1.5, which is interpreted as indifference. Further classification of gonococcal isolates by their susceptibility to ceftriaxone (susceptible versus resistant) did not impact the FICI results. Therefore, auranofin, cannabidivarin, and tolfenamic acid did not show any synergistic or antagonistic activity when used with ceftriaxone.

**TABLE 2:** FICI for auranofin, cannabidivarin, and tolfenamic acid with ceftriaxone against 70 clinical Neisseria gonorrhoeae isolates

Antimicrobial combination/strain group^*a*^	MIC^*b*^ (mg/L), median (range)				FICI,^*c*^ median (range)	Interpretation
	MIC_A_^single^	MIC_A_^combined^	MIC_B_^single^	MIC_B_^combined^		
AUR with CRO						
CRO-S (*n* = 49)	0.5 (0.125 to 1)	0.5 (0.125 to 1)	0.06 (0.002 to 0.125)	0.06 (0.004 to 0.125)	1.50 (0.75 to 3.00)	Indifference
CRO-R (*n* = 21)	0.5 (0.25 to 1)	0.5 (0.25 to 0.5)	0.25 (0.25 to 1)	0.25 (0.25 to 1)	1.50 (1.00 to 3.00)	Indifference
All (*n* = 70)	0.5 (0.125 to 1)	0.5 (0.125 to 1)	0.06 (0.002 to 1)	0.06 (0.004 to 1)	1.50 (0.75 to 3.00)	Indifference
CAN with CRO						
CRO-S (*n* = 49)	8 (4 to 16)	4 (4 to 8)	0.06 (0.002 to 0.125)	0.06 (0.004 to 0.125)	2.00 (0.75 to 3.00)	Indifference
CRO-R (*n* = 21)	8 (4 to 16)	8 (4 to 8)	0.25 (0.25 to 1)	0.125 (0.03 to 1)	1.50 (1.00 to 2.50)	Indifference
All (*n* = 70)	8 (4 to 16)	8 (4 to 8)	0.06 (0.002 to 1)	0.06 (0.004 to 1)	1.50 (0.75 to 3.00)	Indifference
TOL with CRO						
CRO-S (*n* = 49)	8 (2 to 16)	8 (4 to 8)	0.06 (0.002 to 0.125)	0.03 (0.004 to 0.125)	2.00 (0.75 to 3.00)	Indifference
CRO-R (*n* = 21)	8 (2 to 16)	4 (2 to 16)	0.25 (0.25 to 1)	0.25 (0.06 to 1)	1.50 (0.75 to 2.50)	Indifference
All (*n* = 70)	8 (2 to 16)	4 (2 to 16)	0.06 (0.002 to 1)	0.06 (0.004 to 1)	1.50 (0.75 to 3.00)	Indifference

a FICI, fractional inhibitory concentration index; CRO, ceftriaxone; AUR, auranofin; CAN, cannabidivarin; TOL, tolfenamic acid; CRO-S, ceftriaxone-susceptible (MIC ≤ 0.125); CRO-R, ceftriaxone-resistant (MIC > 0.125).

b MIC_A_ is AUR, CAN, or TOL; MIC_B_ is CRO.

c FICI were interpreted using the following criteria: FICI < 0.5: synergy; FICI = 0.5–4.0: indifference; FICI > 4.0: antagonism.

### Analysis of auranofin, cannabidivarin, and tolfenamic acid bactericidal activity.

**FIG 2:**
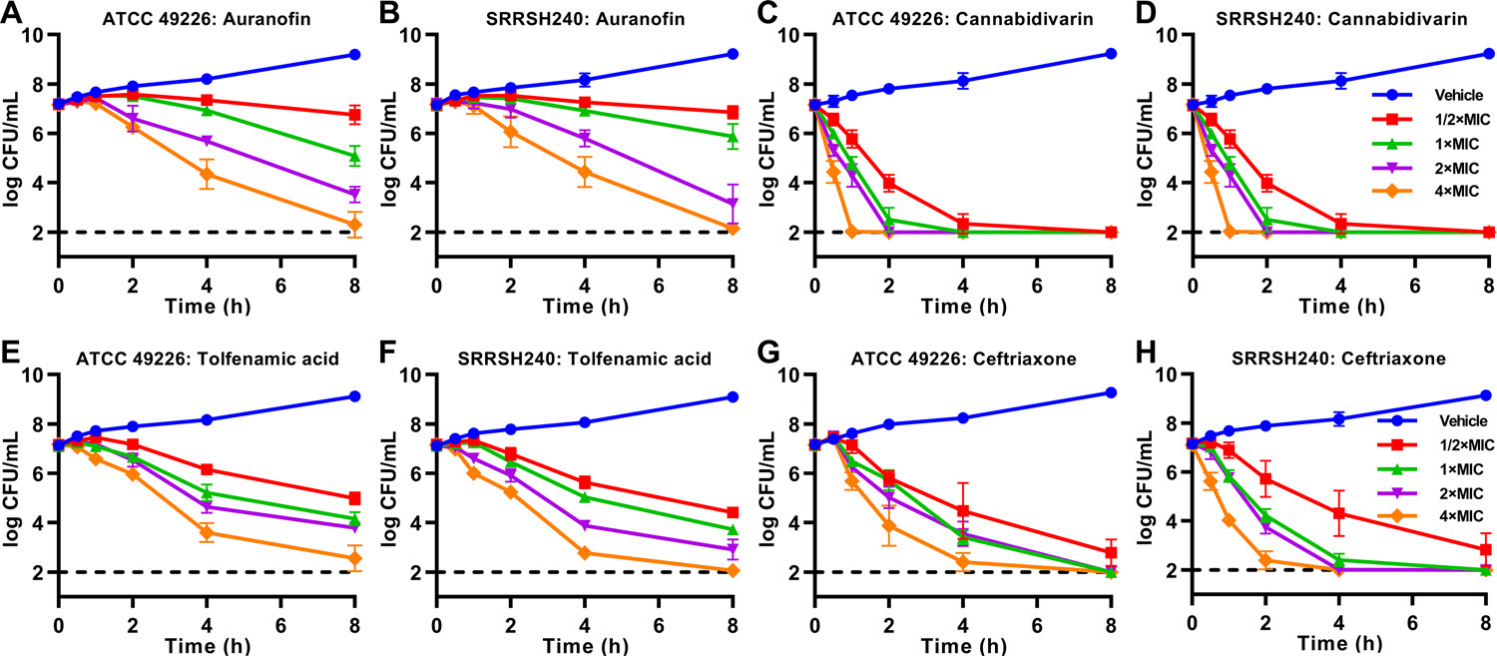
Time-kill assays for auranofin, cannabidivarin, and tolfenamic acid against Neisseria gonorrhoeae. Reference strain ATCC 49226 (A, C, E, G) and FC428-associatedd strain SRRSH240 (B, D, F, H) were exposed to auranofin (A, B), cannabidivarin (C, D), tolfenamic acid (E, F), or ceftriaxone (G, H) at 0.5 × 1×, 2×, or 4× the MIC or the vehicle control (GC broth). Survival curves represent the mean and standard deviation of three biological independent repeats. Auranofin 1 × MIC: 0.5 mg/L; cannabidivarin 1 × MIC: 8 mg/L; tolfenamic acid 1 × MIC: 8 mg/L; ceftriaxone 1 × MIC: 0.008 mg/L for ATCC 49226, 1 mg/L for SRRSH240.

Bactericidal activity of auranofin, cannabidivarin, and tolfenamic acid against N. gonorrhoeae was investigated in time-kill assays. All three compounds were bactericidal against both the ATCC 49226 reference strain and the FC428-associated strain SRRSH240 (Fig. 2). For both strains, auranofin and tolfenamic acid reached approximately 10^5^-fold inactivation at the highest 4 × MIC dose (2 mg/L for auranofin and 32 mg/L for tolfenamic acid) after an 8-h exposure, while cannabidivarin reached over 10^5^-fold inactivation at a 4 × MIC dose (32 mg/L) after only a 1-h exposure. Even at a 0.5 × MIC (4 mg/L) dose, over 10^5^-fold inactivation was obtained after an 8-h exposure to cannabidivarin. Therefore, it appears that particularly cannabidivarin displays very potent bactericidal activity. For reference, ceftriaxone reached over 10^5^-fold inactivation after an 8-h exposure of N. gonorrhoeae to a 1 × MIC dose.

## DISCUSSION

Repurposing of drugs that are approved for other clinical applications could be a fast and economic approach for the development of alternative antimicrobials. Auranofin is a gold-based compound that has originally been approved for treatment of rheumatoid arthritis, but drug repurposing studies have subsequently shown activity as an antibacterial agent (19–21, 27). Auranofin was recently also identified in a drug repurposing screen for antimicrobial activity against N. gonorrhoeae and displayed a MIC of 1 mg/L against all 51 tested clinical isolates (16). In our screen containing 575 multidrug-resistant isolates, the auranofin MIC was mostly in the 0.25 to 0.5 mg/L range, with only nine strains displaying an MIC of 1 mg/L. Auranofin has proven safe for human use and is suitable for oral dosing, with quick absorbance from the gastrointestinal tract, peak plasma concentrations (*Cmax*) of 0.06 to 0.3 mg/L, a half-life of 15 to 35 days, and final excretion through feces (64% to 85%) (28–30). However, these pharmacokinetic data are based on human rheumatoid arthritis dosing in which long-term daily dosing is in the 3 to 9 mg/day range, although modeling of a daily 21-mg dose reached a plasma *Cmax* of 1.4 mg/L after 14 days of therapy (30). Although these pharmacokinetics data suggest that long-term low concentration auranofin dosing could potentially reach gonococcal inhibitory concentrations, clinical applicability of elevated auranofin dosing for treatment of gonococcal infections remains to be determined.

Cannabidivarin is a propyl analogue of cannabidiol, which are cannabinoid derivatives that are widely studied for their influence on pain sensation, memory, and cognition, and for their anticonvulsant, sedative, and immunosuppressive activities (22, 23, 31). Cannabidiol previously showed antimicrobial activity against several Gram-negative bacterial pathogens, including N. gonorrhoeae. Further screening of structural analogues identified the most potent activity against N. gonorrhoeae for cannabidivarin, with an MIC of 0.03 to 0.5 mg/L against reference strain ATCC 19424. In our study, cannabidivarin activity was less potent, with an MIC of 4 to 8 mg/L for most of the clinical isolates. However, bactericidal activity of cannabidivarin appeared particularly strong, making it an interesting compound for further anti-gonococcal studies. Importantly, cannabidivarin and cannabidiol have displayed good safety profiles for human use and absence of psychoactive activity (32, 33). A recent phase I clinical trial for epilepsy therapy in pediatric patients showed that an oral cannbidivarin dose of 2.5 mg/kg resulted in a plasma *Cmax* of 4 to 14 μg/L within 2 h following the dose (34). However, its main metabolic side-product, 7-carboxy-cannabidivarin, reached a plasma *Cmax* up to 3 mg/L within 2 h, which is close to the MIC range of 4 to 8 mg/L observed for the majority of gonococcal isolates in hour study. Because it has previously been shown that 7-carboxy-cannabidivarin also displayed activity against N. gonorrhoeae in the 2 to 16 mg/L MIC range (17), cannabidivarin or its metabolic side-products might be able to reach sufficient *in vivo* levels for antigonococcal activity when dosing is optimized.

Tolfenamic acid is an aminobenzoic acid with anti-inflammatory activity through inhibition of cyclooxygenases involved in prostaglandin production (26, 35). It has also demonstrated anticancer activity and potential to slow development of Alzheimer’s disease (35, 36). Recently, a drug repurposing study tested three fenamic acid compounds—tolfenamic acid, flufenamic acid, and meclofenamic acid—for antimicrobial activity against N. gonorrhoeae (18). Tolfenamic acid showed the strongest activity, with an MIC_90_ of 8 mg/L against a collection of 45 clinical isolates, similar to the observations made in our study. Tolfenamic acid is orally bioavailable and rapidly absorbed from the gastrointestinal tract (35). In healthy volunteers, oral dosing of 800 mg tolfenamic acid resulted in a plasma *Cmax* of 12 mg/L (37), which is higher than the MIC for the majority of tested gonococcal isolates in our study. However, tolfenamic acid shows a half-life of only 2.5 h and a large fraction of absorbed tolfenamic acid is metabolized in the liver (37). Therefore, it is currently unknown whether it can reach sufficient activity at the infected sites for treatment of gonococcal infections.

In conclusion, this study investigated antimicrobial activity of the clinically approved compounds auranofin, cannabidivarin, and tolfenamic acid against a collection of 575 clinical multidrug-resistant N. gonorrhoeae isolates. All three compounds showed consistent antimicrobial activity against all N. gonorrhoeae isolates, including against the high-level ceftriaxone-resistant strains associated with the FC428 clone. Whether these compounds are clinically suitable for antigonococcal therapy remains to be determined in future studies.

## MATERIALS AND METHODS

### Antimicrobial susceptibility testing.

The MIC of all antimicrobial compounds was determined by agar dilution method (38) for 575 clinical N. gonorrhoeae isolates covering the periods 2011 to 2012 (39), 2015 to 2017 (40), and 2019 (11) and N. gonorrhoeae strain ATCC 49226 was included for quality control. Overnight cultured bacteria on GC agar (Oxoid) supplemented with 1% (vol/vol) Vitox (Oxoid) were suspended and approximately 10^4^ CFU was applied onto GC agar plates containing 1% Vitox and antimicrobial agents in 2-fold dilution series. The MIC was defined as the lowest concentration of the compound for which no growth was detected.

### Antimicrobial susceptibility correlation analysis.

Cross-resistance between ceftriaxone and auranofin, cannabidivarin, or tolfenamic acid was analyzed by MIC correlation analysis. The correlation coefficient R was determined by linear regression analysis of log-transformed MIC values. Strong correlation was defined as *R* ≥ 0.4 in GraphPad Prism 8.

### Chequerboard antimicrobial synergy testing.

Antimicrobial synergism between ceftriaxone and auranofin, cannabidivarin, or tolfenamic acid was performed by agar dilution method on GC agar supplemented with 1% Vitox following the chequerboard strategy (41). Synergism was determined for the 70 N. gonorrhoeae clinical isolates covering 2019 (11) and strain ATCC 49226 was included for quality control. MICs were determined for single antimicrobials (MIC_A/B_^single^) and combined (MIC_A/B_^combined^). The fractional inhibitory concentration index (FICI) was calculated as (MIC_A_^combined^/MIC_A_^single^) + (MIC_B_^combined^/MIC_B_^single^). A FICI < 0.5 was defined as antimicrobial synergism; a FICI > 4 was defined as antagonism; a FICI = 0.5 to 4 was defined as indifference.

### Time kill-assays.

Overnight-cultured bacteria on GC agar supplemented with 1% Vitox were suspended at 10^7^ CFU/mL in 12 mL GC broth with 1% Vitox. Ceftriaxone, auranofin, cannabidivarin, or tolfenamic acid were diluted in GC broth and added at 0.5×, 1×, 2×, or 4× the MIC. The vehicle control was GC broth only. Cultures were incubated at 37°C and 200 rpm and samples were collected in a time-series for CFU determination on GC agar with 1% Vitox. Time-kill assays were performed with the N. gonorrhoeae reference strain ATCC 49226 and the FC428-associated clinical isolate SRRSH240 (11).

### Ethical approval.

Ethical approval was not needed for this study.
